# Sudden onset chest pain after a CT-scan of the aorta

**DOI:** 10.1007/s12471-024-01914-3

**Published:** 2024-12-23

**Authors:** Fabienne E. Vervaat, Thomas van Brakel, Sjoerd Bouwmeester

**Affiliations:** 1 Department of Cardiology, Catharina Hospital, Eindhoven, The Netherlands; 2 Department of Cardiothoracic surgery, Catharina Hospital, Eindhoven, The Netherlands

## Answer

**Fig. 1:**
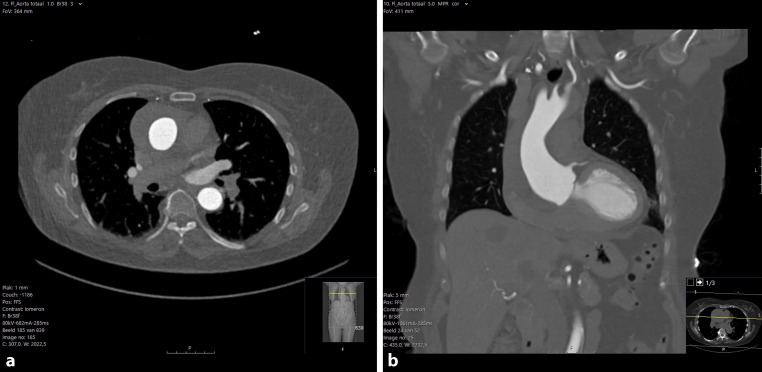
**a**, **b** CT-scan of the aorta after symptom onset showing an intramural hematoma located at the aortic root and ascending aorta

Given the patient’s medical history, there was an immediate suspicion of type A aortic dissection. A new CT-scan was performed and a type A intramural hematoma (IMH) was diagnosed (Fig. 1). Type A IMH is recognized and differentiated from a type A aortic dissection by a crescentic or circular aortic wall thickening in the absence of an intimal flap [1]. IMH is the cause of acute aortic syndrome in 5–25% of the cases with ~30% involving the ascending aorta and in 12% of patients an IMH can evolve into an aortic dissection [1]. The current treatment for type A IMH is comparable to that of type A aortic dissection, which is surgery [1]. Emergency surgery was performed successfully, involving biological aortic valve replacement and hemi aortic arch replacement. The patient’s post-operative recovery was uneventful, and she was discharged after ten days. During the first outpatient follow-up two months later, the patient reported being symptom-free and recovering well.
